# Pesticides Identification and Sustainable Viticulture Practices to Reduce Their Use: An Overview

**DOI:** 10.3390/molecules27238205

**Published:** 2022-11-24

**Authors:** Samuel Tucker, Georgiana-Diana Dumitriu (Gabur), Carmen Teodosiu

**Affiliations:** 1 Department of Environmental Engineering and Management, “Gheorghe Asachi” Technical University of Iasi, 700050 Iasi, Romania; 2 Faculty of Horticulture, Iasi University of Life Sciences, 700490 Iasi, Romania

**Keywords:** pesticides, analytical methods, grapes and wines, sustainable viticulture

## Abstract

The use of pesticides is a necessary practice in the modern era. Therefore, it is impossible to ignore the pesticide market, which has developed into one of the most lucrative in the world. Nowadays, humans are subjected to many potential risks, and significant amounts of toxic compounds enter their bodies through food, drink, and the air itself. Identification and quantification of these hazardous compounds is crucial for the sustainable development of an increasing world population which poses high climatic and political constraints on agricultural production systems. The maximum residue limits for pesticides have been regulated by the Codex Alimentarius Commission and European Union to protect human health. In this review, we have summarized and explained the analytical methods for pesticide extraction and determination. Also, sustainable viticulture practices like organic vineyards, tillage, biopesticides, nanobiopesticides, and precision viticulture are briefly discussed. These new techniques allow wine growers to be more profitable and efficient, while contributing to the reduction of pests and increasing the quality of wines.

## 1. Introduction

Pesticides are described as “anything that prevents, eliminates, or regulates a hazardous organism (‘pest’) or illness, or protects plants or plant products throughout production, storage, and transport” by the European Commission [1]. According to the World Health Organization (WHO) pesticides are considered as a special class of chemical compounds used to destroy a broad range of pests that include weeds, insects, and rodents. The Stockholm Convention on Persistent Organic Pollutants has classed these pesticides as persistent organic pollutants (POPs), and their use is rigorously controlled globally.

**Figure 1:**
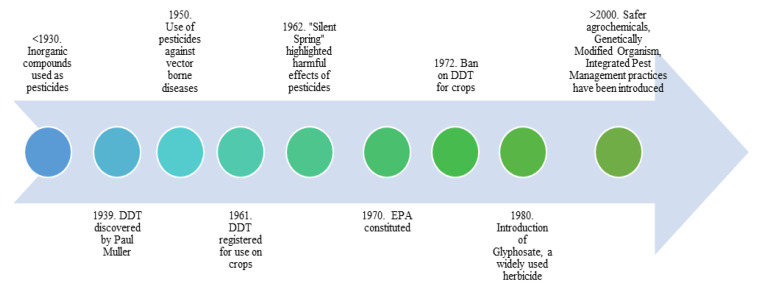
A timeline of pesticide uses since early 1930s.

Pesticides have been used since the Sumerians employed sulphur dust to control insects and mice 4500 years ago, and the Chinese used mercury and arsenic to control lice 3000 years ago [2]. Since then, new discoveries have led to the development of far more effective compounds, and more extensive investigation has resulted in the discontinuation or outright ban of several of these chemicals [3]. Dichloro-diphenyl-trichloroethane (DDT), the first modern synthetic insecticide, is a well-known example of this. During World War II, it was first created to battle malaria, typhus, and other insect-borne infections. After the war, it was widely employed as an insecticide in agriculture, as well as in private homes and gardens, and as a result of its broad use many insects developed resistance to the chemical (Figure 1) [4]. As the use of DDT became more widespread the negative consequences began to emerge, the most serious of which was the significant harm it caused to wildlife populations, particularly birds. DDT-exposed birds lay eggs with abnormally thin shells, raising concerns about the substance’s long and short-term impacts on human health, especially given its potential to persist in soil for up to 15 years following application [3]. As a result, several nations throughout the world began banning DDT in the 1970s, and in 2004 the Stockholm Convention categorized DDT as a “restricted” substance that could only be used against mosquitoes in particular countries to prevent malaria [3].

Pesticides come in a variety of types and classifications, each designed to combat a specific ‘pest.’ Herbicides are the most frequent type and are used to “lower the density of weeds and stimulate the establishment of beneficial species” in agriculture and wilderness environments [5]. Insecticides are used extensively in agriculture and are designed to control, repel, or kill one or more insect species [6]. Fungicides are another form of pesticide that are used to control and remove mold, mildew, and other fungus [7]. Acaricides, nematicides, molluscicides, rodenticides, growth regulators, repellents, and rodenticides [1] are other common pesticides used in a range of industries for a variety of purposes.

## 2. Pesticide Trends around the World

According to the U.N., statistical research predicts, with 95 percent accuracy, that by 2030 there will be between 9.4 and 10.1 billion people on the planet, an additional 2 billion to the population estimated for mid-2019 [8]. The use of pesticides and other agrochemicals is therefore a necessary phenomenon in the modern era, due to their chemical interaction with pests and pathogens that lead to safe, high qualitative vegetables and fruits. In these conditions, it is impossible to ignore the pesticides market, which has developed into one of the most lucrative in the world. Pesticide trade volume in 2018 totalled 5.9 million tons, valuing 37.6 billion USD.

With an increase in pesticide exports from 1,992,898 million tons in 2015 to 2,454,480 million tons in 2019, Asia has recently emerged as the world’s largest exporter of pesticides. China accounted for two-thirds of all pesticide exports, or 1,468,275 million tons, in 2019. Other significant pesticide exporters in 2019 included Germany with 0.48 million tons, India with 0.44 million tons, France with 0.45 million tons, and Belgium with 0.18 million tons. Contrarily, the top three countries importing pesticides in the same year were Brazil with 0.52 million tons, France with 0.26 million tons, and Canada with 0.21 million tons [9].

The application of pesticides to fields is a sign of the various national or local farming practices. The average amount of pesticides used in agriculture around the world increased from 2.28 kg/ha in 2005 to 2.69 kg/ha in 2019, according to FAO [10].

During this time, pesticide use in Asia and the Americas exceeded the global average, rising from 3.18 and 2.89 kg/ha in 2005 to 3.68 and 3.70 kg/ha in 2019, respectively. Europe used an average of 1.66 kg of pesticides per hectare of agricultural land in 2019, with the highest pesticide application rates in the Netherlands (8.88 kg/ha), Belgium (6.96 kg/ha), Montenegro (6.07 kg/ha), Ireland (5.97 kg/ha), Italy (5.21 kg/ha), and France (4.46 kg/ha).

While pesticides have a number of extremely useful attributes, they also have a “*deleterious effect on humans and the environment and their presence in food is particularly dangerous*” [11]. Many pesticides have been found to have severe effects with regards to endocrine disorders, reproductive problems, cancers, diabetes, obesity, and cardiovascular diseases [12].

According to a World Bank report, pesticide poisoning is thought to be the cause of 355,000 annual deaths [13]. According to their mode of action and level of exposure, different pesticides obviously have varying degrees of toxicity in humans and other biota. They consequently have a variety of consequences on living things. According to Muhammad et al. [14] and Sidhu et al. [15] pesticides affect the nervous systems of aquatic and terrestrial fauna and humans, resulting in endocrine, metabolic, and neurological disorders as well as various cancers like leukemia and bladder cancer.

To safeguard human health, while still facilitating world trade, the WHO and the FAO have set up a joint Codex Alimentarius Commission in order to coordinate food standards, as well as establishing universal Maximum Residue Levels (MRLs) of pesticides legally permitted in or on food or animal feed. Unfortunately, the MRLs for a particular pesticide used on a particular commodity can vary from country to country, causing a lack of harmonization in international trade. Farmers will produce food according to their own countries standards but are then penalized when they try to sell their product internationally and it does not meet the required MRLs. In addition, this poses a potential health risk to consumers in countries with stricter MRL standards when importing from a country with lower MRLs [16].

Although there are broad rules for pesticide residues in fruits, vegetables, or drinking water, scarce attention is still dedicated to derivate products, as grape must and grape-based products, which may contain these commodities as an ingredients. The maximum residue limits for grapes along with the acceptable daily intake (ADI), Acute Reference Doses (ARfT) and Acceptable Operator Exposure Level (AOEL) are presented in this review (Table 1) [17].

## 3. Pesticides Extraction and Detection

### 3.1. Pesticide Extraction

**Figure 2:**
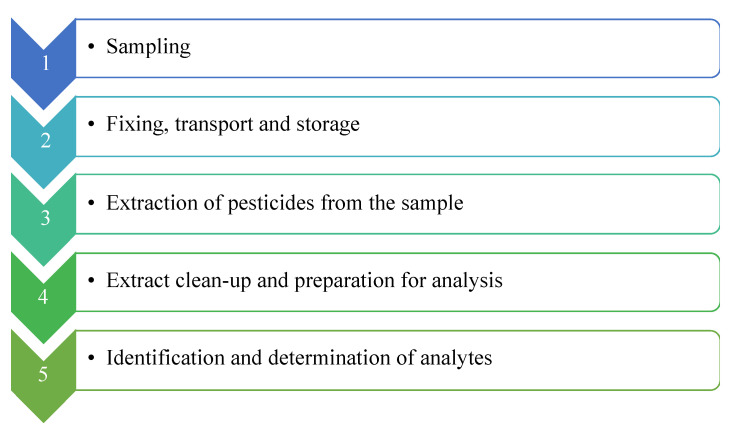
The main stages of analytical procedures for determining pesticides [11].

In general, pesticide determination using analytical methods involves a number of main steps such as: sample preparation, homogenization, extraction, and clean-up procedures including, separation, detection and data analysis. Identification of pesticides residues includes 2 steps: extraction of analytes from the bulk of the samples, and clean-up of the analytes from any co-extractives present in the samples [18]. Figure 2 shows a visual example of this process.

There are many different kinds of extraction techniques, ranging from traditional extraction techniques like the soxhlet extractor and quick, easy, cheap, effective, rugged, and safe (QuEChERS) to microextraction techniques, which are divided by different sorbents, such as the liquid-phase microextraction (LPME), solid-phase microextraction (SPME), and stir bar sorptive extraction (SBSE) [19].

The advancement in analytical procedures brought about by the evolution of extraction techniques has decreased the complexity of sample treatment while simultaneously increasing the accuracy and precision of the analysis [16]. The choice relies on the analytical problem at hand because each technique has advantages and disadvantages of its own.

The Mills method was created in 1963 and is based on the use of acetonitrile to remove organochlorine insecticides and other nonpolar pesticides from low-fat meals. Following extraction, partitioning takes place with the addition of sodium chloride into a nonpolar solvent, such as petroleum. The extract is cleaned using a Florisil column. It should be noted that when analyzed with a nonpolar solvent, moderately polar pesticides like organophosphorous insecticides largely evaporate [16].

The Stoherr method is a small variation of the Mills method that seeks to broaden the procedure’s analytical applicability to compounds with various polarities [16]. Once more, acetonitrile is used for extraction, but this time, Florisil is replaced by acid-treated charcoal and nonpolar petroleum is swapped out for dichloromethane, which has a higher polarity. The vast majority of organophosphorous pesticides found in fruits and vegetables, including grapes, can be removed using this technique.

The Luke method, developed in 1975, centres around acetone as an extractant, little cleaning, and a variety of GC systems with element-specific and element-selective detectors. With this technique, Florisil is used for both the combined cleanup of organochlorine and organophsphorous pesticides. In order to fully saturate the aqueous phase, sodium chloride is also added. This increases the amount of acetone and raises its polarity, which results in excellent polar analyte recoveries [16].

Following on from the Luke Method is liquid-liquid extraction (LLE), also known as solvent extraction and partitioning, considered as a green analytical chemistry method. Even though this method is compatible with the sustainable development concepts, LLE is not employed in multiresidue procedures [16]. This approach, which separates chemicals based on their relative solubility in water and an organic solvent, is typically employed for sample cleanup. Dichloromethane is added to a solvent combination after acetone extraction. This procedure works with grapes and their by-products, but it is time-consuming, labor-intensive, and requires a lot of hazardous solvents that are dangerous to use around people [16]. The limits of detection (LODs) range obtained in one study [20] which combined the LLE method with HPLC-MS/MS analytical systems to determine four pesticides (malathion, diazinon, imidacloprid, and triamedimefon) in fruit juice samples was from 3 × 10^−4^ to 3 × 10^−2^ mg/L, with a correlation coefficient of 0.995. Another study by Farajzadeh et al. [21] devised a straightforward, quick, and affordable approach for pyrethroid pesticide determination using the LLE and dispersive liquid-liquid microextraction (DLLME), with detection limits ranging from 0.02 to 0.17 mg/kg.

An alternative method to LLE, known as solid-phase extraction (SPE), was created in the middle of the 1970s for the separation, purification, preconcentration, and solvent exchange of solutes for solutions. A sample is isolated, concentrated, purified, and cleaned up using this technique. Given that it involves less time, less solvent, fewer stages, and is more cost-effective than LLE, it offers a number of major advantages. Additionally, SPE can be used on materials in conjunction with other analytical techniques to enhance the process; however, the main drawback of this technique is a significant degree of variability in the results [16].

Solid-Phase Microextraction (SPME) was developed in 1990 to further optimize SPE/LLE and redress their limitations. This method requires negligible solvent for sample preparation and uses a fused silica fiber coated with a stationary phase attached to a micro syringe. It is important to note that the extraction temperature, time and ample agitation must be optimized, and operating conditions need to be consistent. The main advantages are the reduction of solvent use, the combination of sampling and extraction into one step, the ability to examine smaller sample sizes, the possibility to use fibers many times without the loss of the adsorbate, the possibility to rerun the analysis of any given sample, and major design changes with respect to chromatographs are not necessary. However, the disadvantages include the fact that there is no way of ensuring a sufficiently broad analytical range in a single analysis, there remain some problems with reproducibility, method optimization problems, low recoveries of analytes, as well as limited volume of stationary phase to be applied to the fiber which can lead to incomplete extraction [16].

Matrix Solid-Phase Dispersion (MSPD) was developed in 1989, and was a modification of SPE based on the use of a sorbent which acts as an abrasive in order to produce a modified opening of the solid matrix, allowing for extraction. It is based on a mixer of fine dispersion of the matrix with a sorbent material such as alumina or silica, and many MSPD procedures use co-columns to obtain further fractionation. Advantages of MSPD include the small amount of sample and solvent, fewer experimental steps, direct handling of samples, ease of implementation, reduced solvent consumption, and the low overall cost. The main disadvantages are the insufficiently wide analytical range of a single sample, its unsuitability for dry samples or samples with a high lipid content, and the fact that it requires an additional cleanup step. A rapid and sensitive multiresidue method for the analysis of pesticides in fruits (acetamipride, carbendazim, carbaryl, carbofuran, imidacloprid, malathion, propazine, dimethoate and tebufenozide was developed by Radisic et al. [22]. The method involves an extraction procedure based on MSPD using diatomaceous earth as a dispersant and dichloromethane as the eluent. In addition, according to European and Brazilian monitoring programs, most of the selected pesticides are frequently detected pesticides in fruits and vegetables, and that MSPD requires approximately 95% less solvent and can be performed in 90% less time when compared to such classical methods.

Stir Bar Sorptive Extraction (SBSE) was developed in 1999 and attempted to overcome the limited extraction capacity of SPME fibers. According to Urkude et al. [16], this method is a solvent-less sample preparation method for extraction and enrichment of organic compounds from aqueous matrices. A large surface area is created by a thick bonded absorbent layer on a glass stirrer bar, leading to a higher phase ration and thus a better recovery and sample capacity. It has a high effectiveness for nonpolar and medium polarity compounds from liquid samples, it is easy to apply and automate, and it is highly flexible, sensitive, reputable, and reproduceable. However its main disadvantage is the fact that it can only be applied to medium-high volatility and medium-high thermo-stability analytes, and sampling times and cost of instrumentation can be high.

In order to separate desired analytes from the sample matrix and introduce them into the solvent, microwave radiation is used in microwave-assisted extraction (MAE). As a result, the solvent may be heated quickly, and extraction typically lasts 15 to 30 min. It has a high sample throughput, uses less solvent, operates at low temperatures, has great automation and extraction rates, and allows for the uninterrupted extraction of multiple samples at once. But only thermally stable chemicals can be used with this method, and they need to be dissolved in a polar solvent like water [16].

QuEChERS is the name of a method in which many pesticides can be analysed simultaneously in different food matrices, and similar to many of the techniques previously outlined, it involves an extraction, separation and cleanup phase. Two predominant methods of QuEChERS arose, the European Committee for Standardization proposed a citrate-buffered method, while Association of Official Analytical Collaboration (AOAC) International proposed an acetate-buffered method. While both were especially effective in terms of lipid coextractives and therefore well suited towards extraction of the high-sugar grape, the acetate-buffered method was concluded to be more appropriate for use with grapes. The main benefits of this QuEChERS include high recoveries with a wide range of polarity and volatility, high sample throughput, the need for simple equipment for sample preparation, the need for a smaller amount of organic solvent, lower reagent costs, ruggedness, and the removal of organic acids and other potential contaminants during cleanup [16]. It also offers a significant advantage over traditional methods which require the use of multistage procedures, large samples, and one or more extract cleanup steps. The number one disadvantage of QuEChERS is that the final extract has to be concentrated to a greater extent in order to achieve the necessary sensitivity and thus to achieve the limits of quantification desired. Many modifications were proposed to the QuEChERS model, including the use of different solvents such as graphitized carbon black, or the use of low-temperature precipitation, which allowed for the extraction of large numbers of pesticides from different classes and matrices, as well as for advanced cleanup stage processes [23,24,25,26].

There are other forms of microextraction methods used for the pesticide detection which include dispersive liquid liquid microextraction (DLLME), single drop microextraction (SDME), continuous flow microextraction (CFME), hollow fiber-liquid phase microextraction (HF-LPME), as well as a combination of SPE, DLLME, and solid liquid extraction (SLE) [18]. While there are a number of different extraction techniques used, each have their own distinct advantages and disadvantages depending on a number of criteria. These advantages and disadvantages are summarized for each technique below in Table 2 according to research carried out by Samsidar et al. [18], and Wilkowska and Biziuk [27].

### 3.2. Pesticide Detection

Chromatography, which has been employed in the detection and analysis of a range of pesticides, consists of a mobile phase (gas or solvent) and a stationary phase, such as a column or capillary tube. Gas chromatography (GC), liquid chromatography (LC), high-performance liquid chromatography (HPLC), and supercritical fluid chromatography (SFC), as well as mass spectrometry (MS), are different types of chromatography based on the mobile phases used [28]. In the future, chromatography and its combination with mass spectrometry will be widely used.

With its beginnings in the 1950s and current widespread use, GC is a significant detection method. In order to assess the sample’s composition after gasification, the inert gas is transferred into the separation apparatus. For non-polar, highly volatile, and quickly vaporized chemicals, the GC technique is appropriate. To estimate pesticides using the GC method, experts from all around the world have recently used a variety of extraction techniques.

Many compounds in plant-derived food that are rarely studied or difficult to identify, like highly polarized and non-volatile and/or thermally labile pesticides, can be quickly and effectively identified using the liquid chromatography-mass spectrometry (LC-MS) method, even those that are not GC-amenable. It is now possible to detect pesticide traces in complex systems like fruits and vegetables, grains, and animal-derived foods thanks to better LC-MS/MS.

In HPLC, the solvent moves under high pressure that is generated by a pump to get around the pressure drop challenge and shorten the separation time. In terms of pesticide detection, HPLC-MS technology has produced a wealth of qualitative and quantitative data. According to Bletsou, Jeon, Hollender, Archontaki, and Thomaidis [29], HPLCQqQ-MS in multiple reaction monitoring (MRM) has demonstrated great sensitivity, selectivity, and low detection limits for studies.

With advantages in speed, sensitivity, and low cost, the supercritical fluid chromatography-tandem mass spectrometry (SFC-MS/MS) method is frequently used for separations involving non-volatile or thermally labile pesticides as well as to quantify chiral or achiral chemical compounds in biological samples [30].

The most used method for detecting pesticide residues in food samples generated from plants is LC or GC combined with MS. However, these methods necessitate specialized equipment used by skilled individuals, which is quite expensive and hostile to the environment as many chemical agents may be consumed during the detection [31]. Spectrum analysis is more effective than chromatography methodology at detecting pesticide residues due to its high sensitivity and quick process.

Spectrum analysis is a complementary chromatography method that is effective for detection of pesticide residues due to its high sensitivity and quick process. The most popular spectroscopic techniques are based on the Raman spectrum, near-infrared spectroscopy, and fluorescence spectrum. Resonance Raman spectroscopy (RRS), coherent anti-Stokes Raman spectroscopy (CARS), stimulated Raman spectroscopy (SRS), surface-enhanced Raman spectroscopy (SERS), and tip-enhanced Raman spectroscopy (TERS) are some of the most advanced Raman spectroscopy techniques available today [32].

The pesticide detection process calls for significant human resources and intricate pre-treatment techniques. Numerous quick detection techniques for pesticide residues have been created in such situations, making it simple, quick, and accurate to check pesticide residues. Some sensors, such as electrochemical and optical methods, can measure pesticides with adequate accuracy and over the proper time. The efficacy of electrochemical sensors, which use working electrodes as a transducer, depends on the analyte’s potential redox state and the working potential. Nanosensors have been proposed for the detection of pesticides as a result of the development of nanotechnology. With carbon nanotubes as an example, the development of nanosensors has increased their sensitivity, stabilizing the effect on suppressing acetylcholine esterase (AChE) activity. Gas chromatography (GC), gas chromatography mass spectrometry (GC/MS), and gas chromatography tandem mass spectrometry (GC/MS/MS) are often used because of their high separation power, selectivity, and identification capabilities of MS.

Different studies have outlined the effectiveness of LC-MS/MS and GC/MS respectively [33]. In addition, the various sensitive detectors coupled with GC such as a nitrogen phosphorus detector (NPD), a flame ionization detector (FID), a flame photometric detector (FPD), and an electron capture detector (ECD), have improved the detection and quantification of pesticides, with ECD being especially useful for the organochlorine pesticides, NPD for organophosphorus and nitrogenated pesticides, and FPD for sulpher and phosphorous pesticides [16]. Enzyme-linked immunosorbent assay (ELISA) is another conventional method and due to its reliable high-throughput immunoassay it is currently known as the most prevalent form of immunoassay for pesticide monitoring tools. Capillary electrophoresis (CE) is another valuable analysis technique and relatively applicable for various practices due to the fact that it requires small volumes of reagents and samples, and has great separation efficiency [18].

## 4. Vineyard and Wine and Specific Research on Pesticides

In research undertaken by Nieto-Garcia et al. [34], GC-QqQ-MS/MS was used to optimize a new approach for determining pesticide residues at trace levels in dietary supplements from grape seed extracts. Because of the matrix’s complexity, numerous cleansing stages must be included in the extraction operation to eliminate interferences and coextractive compounds, hence improving sensitivity and reducing GC maintenance. In this regard, it was discovered that using a single sorbent is insufficient to provide satisfactory results, and that a combination of sorbents should be utilized instead. The validation criteria (intra-day and inter-day precision, recovery, linearity, limits of quantification (LOQs), and LODs) were assessed and appropriate results were obtained. Given that there is still no European legislation on pesticide residues in nutraceuticals, the LODs and LOQs were deemed adequate.

He et al. [35] proposed an analytical method for wine samples which provides a broad pesticide screen and quantification methodology. Through multiple reaction monitoring (MRM) and isotope dilution analysis mass spectrometry (IDA-MS/MS) collection, liquid chromatography-tandem quadrupole-linear ion trap (LC-QqLIT MS) was used to analyze and screen target pesticides. Pesticides and other organic contaminants were screened for both target and non-target pesticides and other organic contaminants using LC-QTOF MS in an automated IDA-MS/MS. For target and non-target screening and quantification of pollutants, the combination of LC-QTOF MS and LC-QqLIT MS proved to be superior. The combination of both methodologies yielded excellent results in terms of precise quantification and unambiguous confirmation. Quantification was done using an LC-QqLIT MS in MRM mode, which was used as a supplement to LC-QTOF MS quantification at low concentrations. LC-QqLIT MS working in EPI mode and LC-QTOF MS operating in IDA-MS/MS mode, respectively, provided unequivocal detection of target and non-target pollutants. This technology for wine quality control was made feasible and efficient by direct injection of wine samples and wide-ranging contaminants screening combined with MRM measurement. In environmental science and food chemistry, this technology provides a new perspective on pesticide and other contamination screening and quantification.

Vaquero-Fernandez et al. [36] presented a simple, rapid method for the determination of pyrimethanil during the winemaking process from grape to bottled wine. Gas chromatography with nitrogen–phosphorus detection (GC-NPD) was used to make the determination, which was later verified by gas chromatography/mass spectrometry (GC/MS). For different portions of the fruit, the overall process included three methods: surface, skin, and pulp. After a short sample extraction, the proposed SPE-GC-NPD approach permitted rapid determination of pyrimethanil, suitable for monitoring the fungicide in must and wine from red grapes. The procedures for grapes, must, fermenting must, and wine were highly sensitive and offered good recoveries, linearity, precision, and accuracy. The quantification was done with a matrix-matched calibration to avoid matrix effects.

In a paper presented by Pérez-Ortega et al. [37], a generic sample treatment approach based on solid-phase extraction (SPE) using polymeric-type SPE cartridges was developed for large-scale simultaneous assessment of multiclass pesticides and mycotoxins in wines. The sample treatment procedure was evaluated using a liquid chromatography electrospray time-of-flight mass spectrometry method with 60 representative multiclass pesticides and 9 mycotoxins. The results in terms of sensitivity, extract cleanliness, and matrix effects were comparable to earlier studies, with good recovery rates obtained for several pesticide and mycotoxins classes, demonstrating the adaptability and broad applicability of the suggested technique. The method was successfully applied to the analysis of 24 red wine samples acquired on the open market in Spain. Aflatoxin B2 and metalaxyl were the most detected compounds, in 75% and 50% of the studied samples, respectively.

Navarro et al. [38] outlined a rapid multiresidue gas chromatographic method using both electron capture detector (ECD) and nitrogen phosphorus detector (NPD). After a simple extraction of the sample, the suggested approach allows for the rapid assessment of 17 fungicides often employed in vineyards, and may be utilized for their determination in grapes, must, and wine, according to quality control and Good Laboratory Practice (GLP) requirements. Because chromatograms of untreated grape, must, and wine samples are free of interfering peaks, no clean-up is required. The linearity regression coefficients were all at least 0.994. The percentage of spiked grapes, must, and wine samples recovered varied from 78 to 107 percent, with relative standard deviations of less than 14 percent. Individual detection limits ranged from 0.02 to 0.1 ng. Quantification levels ranged from 0.01 to 0.05 mg/kg, all of which were lower than the maximum residue limits imposed by the European Union’s principal wine-producing countries, Spain, France, and Italy. The quantification limits only coincide with the maximum residue limits (0.05 mg/kg) specified by Spanish legislation for fludioxonil and hexaconazole.

A study carried out by Pelajic et al. [39] used GC/MS to establish a new multiresidue approach for determining 25 pesticide residues in red wine. Solid phase extraction was employed to extract samples from wine, with a washing solution of methanol and water, and elution solvents of acetonitrile and n-hexane. For most pesticides, the LOQs were much below 10 lg/L, and recoveries ranged from 70 to 120 percent. Pesticides were detected in 30 of the 32 red wine samples from Croatia, with a total of 15 pesticides discovered, seven of which were at high concentrations.

González-Rodríguez et al. [40] outlined a specific and sensitive method based on ethyl acetate/hexane extraction followed by SPE clean-up with GCB/PSA followed by GC-ITMS and LC-DAD identification for the analysis of fungicides tested in samples (grapes, musts, pomaces, lees, distilled spirits, and wines). Fungicide concentrations in grapes harvested at the legal preharvest time were lower than the EU MRL values; however, new fungicide concentrations in grapes, present in phytosanitary treatments to control downy mildew applied under critical agricultural practices, were higher or close to the EU MRL values. The dissipation of fungicide residues observed during all steps of the white wine-making process was possible, with the pressing and settling stages being the most important in their removal. Except for valifenalate, each fungicide had a very high decrease rate (ranging from 90% to 99%). Estimated MRLs for white wines were proposed for future EU legislation to restrict the level of fungicides in wines based on data acquired during the vinification process.

Recentlly, Yang et al. [41] showcased a novel, simple, and successful method of using Ultra Performance Liquid Chromatography Mass Spectrometry (UPLC-MS/MS) to simultaneously assess the presence of pyraclostrobin, dimethomorph, cymoxanil and cyazofamid in grapes. The kinetics of fungicide degradation and terminal residue levels in grapes using field tests in Zhejiang Province and Tianjin in 2017 was investigated, finding half-lives in grapes ranging from 0.9 to 13.3 days. Cymoxanil degraded the fastest of all fungicides, and the maximum grape terminal residue levels for pyraclostrobin, dimethomorph, cymoxanila and cyazofamid observed during three monitoring intervals were all below the respective MRLs in China.

González-Rodríguez et al. [42] utilized gas chromatography with an ion trap mass spectrometry detector (GC–IT MS) to determine the presence of tebuconazole residues in grapes, musts, and wines. Tebuconazole remained on the solid matter (cakes and lees) as well as the clarifying agent. Tebuconazole was removed from 86 percent of the finished wine. According to these findings, the MRL for tebuconazole in red wines might be set at eight times lower (0.25 mg/L) than the MRL for wine grapes (2 mg/kg). Tebuconazole did not alter alcoholic or malolactic fermentations in vitro, according to the results of in vitro tests. At the same time, neither the degradation nor the adsorption of tebuconazole was affected by these two fermentative processes.

In a study carried out by Heshmati et al. [43], the QuEChERS extraction method was developed and validated in conjunction with GC-MS/MS to assess penconazole, hexaconazole, diazinon, ethion, and phosalone in grapes. The half-life of triazole fungicides was shown to be longer than that of phosphorus compounds in dissipation experiments, which could be a contributing cause to the pesticides’ high preharvest interval (PHI). Meeting spraying criteria in vineyards, such as setting a controlled dose for these pesticides and paying attention to their PHI, can have a major impact on residual pesticides in grapes. The PHI of penconazole, hexaconazole, diazinon, ethion, and phosalone concentration in grape was 15, 23, 12, 13 and 15 days after spraying, according to the current study results. In addition to taking pesticide PHI into account, immersing grapes in a sodium bicarbonate solution could considerably limit pesticide exposure for consumers.

In research carried out during the winemaking process, the fate of zoxamide and its enantiomers was studied in depth by Pan et al. [44]. After each processing method, including washing, peeling, fermentation, and clearing, the enantiomers of zoxamide were separated and identified using ultra-high-performance liquid chromatography coupled with tandem mass spectrometry (UHPLC–MS/MS). All three treatments showed significant enantioselectivity, and the results showed that R-zoxamide deteriorated faster than S-zoxamide during the fermentation process. Each procedure’s processing factors (PFs) were frequently less than 1, and the total process’s PF ranged from 0.019 to 0.051, indicating that the entire process may significantly reduce zoxamide residue in red and white wine. The findings could aid in more precise zoxamide risk assessments during the winemaking process.

Paya et al. [45] set out to determine the in vitro bioavailability of pesticides that control and inhibit insect growth–flufenoxuron, lufenuron, pyriproxyfen, and fenoxycarb–in grapes grown under good agricultural practice (GAP) while adhering to pre-harvest intervals (PHI) for critical conditions (CAP) in the most unfavonurable conditions. In order to determine matrix-related variations, the bioavailability of wines made from grapes was investigated in each test and in standard solutions. The human gastric, intestinal, and absorption processes were replicated. The researchers employed porcine pepsin, pancreatin, bile salts, and semipermeable cellulose dialysis tubing. The residues of the pesticides investigated were extracted using the QuEChERS technique, and the determination was done using HPLC-MS. Fenoxycarb (3.27 percent) and pyriproxifen (2.04 percent) in wine had the highest percentages of dialyzation for grape and wine matrices.

In research carried out by Cus et al. [46], pesticide residues in the vinification process of two white and two red grapevine varietals were monitored. During the vinification process, crushed grapes, cake, must, lees, and wine were all sampled. During the ripening period, grapes were also sampled. All of the samples were taken in triplicate and tested for 117 pesticides. Three internal analytical methods were used to determine pesticide residues: the multi-residual GC-MS method (71 pesticides), the multiresidual LC-MS-MS method (45 pesticides), and the GC-MS method for dithiocarbamate determination. During ripening, the insecticides boscalid and phosalone were the most persistent. Separations during the solid and liquid phases of the vinification process, particularly the pressing of crushed grapes and wine racking following alcoholic fermentation, considerably reduced pesticide residual quantities in must and wine. Boscalid, cyprodinil, dimethomorph, fenhexamid, metalaxyl, and procymidone were the most persistent pesticides in grapes during ripening.

Golge and Kabak [47] examined the levels of 172 pesticide residues in table grapes in Turkey from August to October 2016. 280 table grape samples were collected from supermarkets, bazaars, and greengrocer shops throughout four Turkish provinces. Liquid chromatography with tandem mass spectrometry was used to examine the samples. Quantification limits varied from 0.002 to 0.010 mg/kg. The validation data demonstrated good recoveries, repeatability, and reproducibility, as well as meeting the rest of the European SANTE/11945/2015 Guideline’s standards. In 59.6 percent of the table grapes, pesticide traces were discovered. 20.4 percent of the samples had residues over the EU limit residue values. Azoxystrobin, chlorpyrifos, boscalid, and cyprodinil were the most common pesticide residues. Lower bound, middle bound, and upper bound values were replaced for left-censored results (40.4 percent of the results). The hazard index (HI) for adults was 3.37 percent and 9.42 percent for children in the worst-case scenario. Chlorpyrifos was the leading cause of HI (65 percent).

In another study undertaken by Castro et al. [48], liquid chromatography with tandem mass spectrometry using triple quadrupole (QqQ) and quadrupole time-of-flight (QTOF) MS instruments was used to investigate the coexistence of the anilinopyrimidine fungicides pyrimethanil (PYR), cyprodinil (CYP), and suspected metabolites in wine samples. For the first time, quantitative data acquired from wine samples after solid-phase extraction (SPE) revealed the systematic existence of 4-hydroxyanilino derivatives of PYR and CYP in wines bearing parent fungicide residues at concentrations ranging from 0.2 to 58 ng/mL. Red wines had higher concentration ratios (hydroxylated derivative/active fungicide) than white wines, especially in the case of PYR. PYR-4OH concentrations were twice as high as PYR concentrations in red wines on average. In the structure of both anti-botrytis fungicides, a targeted search of hydroxyl derivatives in wine extracts using LC-QTOF-MS revealed the presence of additional hydroxylation locations in the pyrimidine ring and/or in the alkyl substituents bonding to this cycle. Furthermore, free and glycosylated forms of both fungicides’ hydroxylated metabolites coexist in wine samples. This research established that hydroxylated and glycosylated metabolites are present in grapes prior to vinification in the case of CYP.

In a study published in 2006, Pose-Juan, Cancho-Grande, Rial-Otero, and Simal-Gándara [49] examined the rates of degradation of four drugs in grape juice: cyprodinil, fludioxonil, procymidone, and vinclozolin. These pesticides were removed using a dichloromethane/acetone solution (4:1, v/v, 75 mL), and their identities were ascertained using gas chromatography mass spectrometry (GC/MS).

## 5. Sustainable Viticulture Practices to Reduce Pesticides Use

- No-tillage

**Figure 3:**
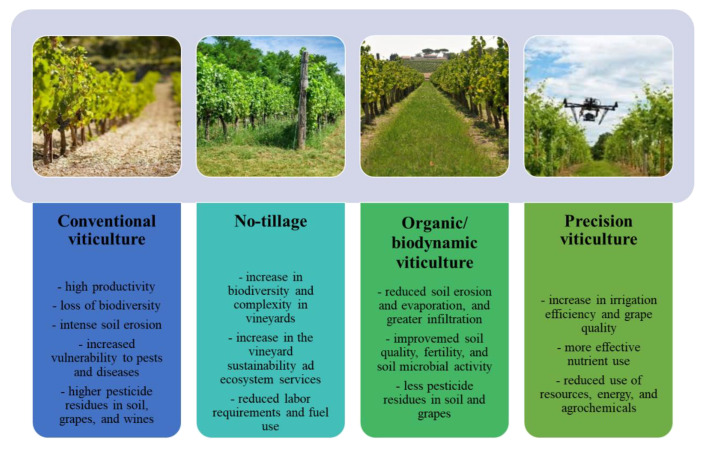
Sustainability of viticulture practices.

In perennial agroecosystems such as vineyards, tillage has been shown to decrease plant [50,51] and animal diversity [52]. Tillage and non-chemical weed control (harrowing, mulching), nutrient application, and other interventions affect soil functioning to varying extents Capowiez et al. [53] (Figure 3). In response to over soil erosion and export of agro-chemicals which become more acute, more farmers have adopted conservation practices including no-till. In the USA, the rate of no-till adoption has grown from 26% in 1990 to 41% in 2008, while conventional tillage has decreased from 49 to 37% during that same period.

- Organic vineyards

Organic viticulture is a production method that underwent significant expansion at the end of the 20th century and has continued to grow ever since. Organic pest management primarily focuses on enhancing the presence of beneficial arthropods to the detriment of pests, using economical and low-impact practices that consider the ecosystem [54]. Organic farming consists of a low-input agro-ecosystem in which crop productivity is based on the natural availability of plant nutrients, the use of green manure and biological pathogen control. Biological control may be realized in various ways such as classical biological control, augmentation, and conservation. Biological control in organic viticulture obtains good results in controlling pests through the use of periodic discharges of biological control agents (augmentation), and by using ecosystem management techniques (conservation). Physical control methods refers to the elimination of insect pests through the application of physical barriers such as nets [55] and kaolin clay [56,57]. Semiochemicals such as pheromones and kairomones attract insects and have a high insect specificity.

In 2019, 63 countries engaged in organic viticulture and certified organic vineyards summed a surface area estimated at approximately 454 kha, or 6.2% of global vine cultivated area [58]. Among all the countries containing organic vineyards, 10 countries exploit 91% of organic vine surfaces, and while only 3 of these 10 countries are European, namely Spain, Italy, and France, EU countries account for 75% of the world’s certified organic vineyard surface area.

- Biodynamic vineyards

Biodynamic agriculture was developed in the 1920s based on a set of conferences performed by the philosopher Rudolf Steiner [59]. This type of agriculture considers a holistic approach concerning the exploitation of natural resources, taking into consideration the sustainability of different elements, such as the crops themselves, animal life preservation, or the maintenance of a high-quality soil, in order to recover, preserve, or improve ecological harmony [60]. By significantly reducing the number of external inputs into the production system, utilizing set preparations to apply to crops which aid fertilization, and the additional application of other homeopathic treatments derived from infusions or plant extracts, this perspective can be achieved. Villanueva-Rey et al. [61] analysed biodynamic viticulture from a life-cycle perspective, and compared it with two other types of viticulture techniques: conventional viticulture and biodynamic-conventional viticulture. The obtained results do not only confirm prior findings that the environmental impact linked to a specific viticulture surface can have relevant variations on an inter annual basis, but also demonstrate strong variability between viticulture practices. In fact, biodynamic viticulture, and to a lesser extent, intermediate biodynamic-conventional vineyards, showed substantially lower environmental profiles for all the environmental impacts assessed.

- Biopesticides and nanobiopesticide

Pesticides that are naturally created by living things like bacteria, herbs, plants, etc. are referred to as biopesticides [62]. Since they are less hazardous to living systems, they are generally safer to employ than synthetic pesticides. The application of pesticides is essential for good crop production since pest infestations in agricultural fields significantly harm crops. Because of the high cost and ongoing usage of synthetic pesticides which has resulted in insect resistance, these chemical substances are no longer effective. *Bacillus thuringiensis* is one of the microbes that has been used to combat several insect pests. The plant *Azadirachta indica* has been discovered to be a powerful pesticide with anti-carcinogenic qualities [63].

Since they are not intentionally generated anywhere, biopesticides are biodegradable. These organic compounds successfully eradicate the intended pest and offer a variety of additional advantages [64,65]. They can increase the nutrients that are available to plants in the soil, and can support plant drought tolerance. Consequently, they are a crucial component of integrated pest control (IPM) techniques. For example, fungi like *Beauveria bassiana* are used in place of insecticides [66]. The amount of pollution produced by using these natural pesticides is minimal.

- Precision Viticulture

Crop monitoring and pesticide spraying are very important aspects within precision agriculture. New autonomous aerial and land vehicles in the near future could result in significant benefits to Agriculture 4.0. The drone was initially created as a military device, including Unmanned Aerial Vehicles (UAVs), Flying Mini Robots and Miniature Pilotless Aircraft. However, the utilization of UAVs in recent years is expanding quickly in agribusiness [67]. These devices incorporate the use of cameras and sensors, and can be grouped into three types: Fixed-wing, Helicopter, and Multi-copter. Semi-controlled drones have been combined with artificial intelligence (Al) in order to monitor farms, which is a remarkably useful device for real-time data analysis. Drones can carry out soil and crop health monitoring scans, as well as assist in irrigation, fertilizer application, and estimate farming yield [68] (Figure 3).

In traditional pesticide application, a manual mechanical sprayer is used which comes with many disadvantages, such as: environmental pollution, less area coverage, increased chemical use, farm labour shortages, lower spray uniformity, and higher costs. Moreover, manual spraying can significantly affect human health through hypersensitivity, asthma, cancer and other diseases [69]. Therefore, it is necessary to improve these deficiencies through the use of the modern drone-mounted sprayer. The advantages of drone use numerous, including: enhanced coverage ability, faster and more straighforward spraying application, increased chemical effectiveness, and the ability to access areas which mechanical sprayers cannot access. One of the most researched and widespread precision technologies is Variable-rate application (VRA), which, through its combined use with Global Positioning Systems (GPS), Geographic Information Systems (GIS), soil sampling, and integrated pest management (IPM), can greatly increase fertilizer input efficiency. It can be applied to seeding, weed and pest control, lime distribution, and fertilizer application [70].

## 6. Conclusions

The monitoring of pesticides has received significant attention over the years due to their toxic effects to both horticultural crops and human safety. Therefore, several techniques have been developed for the extraction and determination of pesticide residues. As this review demonstrates, a wide range of pesticide extraction methods are available, including: liquid-liquid extraction (LLE), solid phase extraction (SPE), solid phase micro extraction (SPME), matrix solid phase dispersion (MSPD), stir bar sorptive extraction (SBSE), microwave-assisted extraction (MAE), Quick, Easy, Cheap, Rugged, Effective and Safe (QuEChERS), etc. Analytical techniques such as gas chromatography or liquid chromatography in conjunction with mass spectrometry (GC-MS, or LC-MS), high-performance liquid chromatography (HPLC), and supercritical fluid chromatography (SFC), are the most frequently used in order to quantify pesticide residues.

Nowadays, the identification and quantification of pesticide residues in grapes and wines is generally carried out by the QuEChERS method and validated in conjunction with gas chromatography/mass spectrometry (GC-MS/MS) and ultra performance liquid chromatography/mass spectrometry (UPLC-MS/MS).

In addition, sustainable management practices to improve vineyard performance within a more sustainable farming system were considered. However, this field of research remains largely unexplored, despite the potential positive effects on vine growth and productivity. Among sustainable viticulture practices, dedicated fertilizers, precision agriculture, and ad-hoc policies will invariably shape the future of this economical area.

## Figures and Tables

**Table 1:** Maximum residue limit (MRL) in grapes [table adapted from ref. [17]].

Pesticide	MRL mg/kg	Year of Adoption	EU Pesticides Data			Regulation European Union, European Food Safety Authority Joint Meeting on Pesticide Residues
			ADI (mg/kg bw/day)	ARfT (mg/kg bw)	AOEL (mg/kg bw/day)	
Acetamiprid	0.5	2012	0.025	0.025	0.025	Reg. (EU) 2018/113
Aldicarb	0.2	1997	Not approved Reg. (EC) No. 1107/2009			
Ametoctradin	6	2013	10.0		2.0	EFSA 2012
Amitrole	0.05	2004	0.001	0.015	0.001	EFSA 2015
Azocyclotin	0.3	2006	0.003	0.02		JMPR 2005
Azoxystrobin	2	2009	0.2		0.2	EFSA 2010
Benalaxyl	0.3	2010	0.04	0.5	0.05	Reg. (EU) 2020/1280
Benzovindiflupyr	1	2017	0.05	0.1	0.04	Reg. (EU) 2016/177
Bifenazate	0.7	2007	0.01		0.0028	05/58/EC
Bifenthrin	0.3	2016	0.015	0.03	0.0075	Reg. (EU) 2018/291
Boscalid	5	2010	0.04		0.1	08/44/EC
Bromopropylate	2	1997	Not approved Reg. (EC) No. 1107/2009			
Buprofezin	1	2010	0.01	0.5	0.04	Reg. (EU) 2017/360
Captan	25	2008	0.1	0.3	0.1	Dir 07/5, SCoFCAH July 08
Carbendazim	3	2008	0.02	0.02	0.02	Dir 06/135
Chlormequat	0.04	2018	0.04	0.09	0.04	EFSA 08
Chlorothalonil	3	2011	0.015	0.05	0.003	Reg. (EU) 2019/677
Chlorpyrifos	0.5	2003	-	-	-	
Chlorpyrifos-Methyl	1	2010	-	-	-	
Clofentezine	2	2008	0.02		0.01	Dir 08/69
Clothianidin	0.7	2012	0.097	0.1	0.1	06/41/EC
Cyazofamid	1.5	2016	0.17		0.3	03/23/EC
Cycloxydim	0.3	2013	0.07	2.0	0.1	EFSA 10
Cyflumetofen	0.6	2015	0.17		0.11	Reg. (EU) No. 2019/716
Cyhexatin	0.3	2006	0.003	0.02		JMPR 2005
Cypermethrins (including alpha- and zeta-)	0.2	2009	0.05	0.2	0.06	Dir 05/53
Cyprodinil	3	2005	0.03		0.03	Dir 06/64
Deltamethrin	0.2	2004	0.01	0.01	0.0075	Dir 03/5
Dichlobenil	0.05	2015	Not approved Reg. (EC) No. 1107/2009			
Dichloran	7	2004	0.005	0.025	0.005	EFSA 10
Difenoconazole	3	2014	0.01	0.16	0.16	Dir 08/69
Dimethomorph	3	2015	0.05	0.6	0.15	Dir 07/25
Dinotefuran	0.9	2013	-	-	-	
Dithiocarbamates	5	2005	-	-	-	
Emamectin benzoate	0.03	2012	0.0005	0.01	0.0003	EFSA 2012
Ethephon	0.8	2016	0.03	0.05	0.03	Dir 06/85, SCoFCAH December 08
Etofenprox	4	2013	0.03	1.0	0.06	EFSA 08
Etoxazole	0.5	2011	0.04		0.03	Reg. (EU) 2020/2105
Famoxadone	2	2005	0.006	0.1	0.0024	Reg. (EU) 2021/1379
Fenamidone	0.6	2015	-	-	-	
Fenarimol	0.3	1999	0.01	0.02		Dir 06/134
Fenbuconazole	1	1999	0.006	0.3	0.02	EFSA 10
Fenbutatin Oxide	5	1995	0.05	0.1		EFSA 10
Fenhexamid	15	2006	0.2		0.2	Reg. (EU) 2015/1201
Fenpyrazamine	3	2018	0.13	0.3	0.2	Reg. (EU) No. 595/2012
Fenpyroximate	0.1	2018	0.01	0.02	0.005	EFSA 2013
Fluazifop-p-butyl	0.01	2017	-	-	-	
Flubendiamide	2	2011	0.017	0.1	0.006	EFSA 2013
Fludioxonil	2	2006	0.37		0.59	Dir 07/76
Flumioxazin	0.02	2016	0.009	0.05	0.018	02/81/EC
Fluopicolide	2	2010	0.08	0.18	0.05	2010/15/EU
Fluopyram	2	2011	0.012	0.5	0.05	Reg. (EU) No. 802/2013
Flupyradifurone	3	2017	0.064	0.15	0.064	Reg. (EU) 2015/2084
Flusilazole	0.2	2008	0.002	0.005	0.005	Dir 06/133
Flutriafol	0.8	2013	0.01	0.05	0.05	11/42/EU
Fluxapyroxad	3	2016	0.02	0.25	0.04	EFSA 12
Folpet	10	2006	0.1	0.2	0.1	Dir 07/5, SCoFCAH July 08
Fosetyl Al	60	2018	-	-	-	
Glufosinate-Ammonium	0.15	2013	0.021	0.021	0.0021	Dir 07/25
Haloxyfop	0.02	2011	0.00065	0.075		EFSA 06
Hexythiazox	1	2010	0.03		0.009	11/46/EU
Imidacloprid	1	2004	0.06	0.08	0.08	Dir 08/116
Indoxacarb	2	2006	0.006	0.125	0.004	06/10/EC
Iprodione	10	-	0.02	0.06	0.04	Reg. (EU) 2017/2091
Kresoxim-Methyl	1.5	2019	0.4		0.9	99/1/EC
Malathion	5	2006	0.03	0.3	0.03	Reg. (EU) 2018/1495
Mandipropamid	2	2009	0.15		0.17	EFSA 2018
Meptyldinocap	0.2	2011	0.016	0.12	0.008	Reg. (EU) No. 1330/2014
Metalaxyl	1	-	0.08	0.5	0.08	2010/28/EU
Methidathion	1	1999	0.001	0.01		JMPR 1997
Methomyl	0.3	2009	0.0025	0.0025	0.0025	EFSA 06
Methoxyfenozide	1	2005	0.1	0.1	0.06	Reg. (EU) 2019/158
Metrafenone	5	2015	0.25		0.43	07/6/EC
Myclobutanil	0.9	2015	0.025	0.31	0.03	EFSA 10
Oxathiapiprolin	0.9	2017	0.14		0.04	Reg. (EU) 2017/239
Parathion-Methyl	0.5	2004	Not approved Reg. (EC) No. 1107/2009			
Penconazole	0.4	2017	0.03	0.5	0.03	Dir 09/77
Permethrin	2	-	Not approved Reg. (EC) No. 1107/2009			
Phosmet	10	1999	0.01	0.045	0.02	Dir 07/25
Propargite	7	2004	0.03	0.06		EFSA 2018
Pyraclostrobin	2	2006	0.03	0.03	0.015	04/30/EC
Pyrimethanil	4	2008	0.17		0.12	Dir 06/74
Quinoxyfen	2	2007	0.2		0.14	Directive 2004/60/EC
Saflufenacil	0.01	2012	Not approved Reg. (EC) No. 1107/2009			
Spinetoram	0.3	2013	0.025	0.1	0.0065	EFSA 2013
Spirodiclofen	0.2	2010	0.015		0.009	EFSA ’09
Spirotetramat	2	2009	0.05	1.0	0.05	EFSA 2013
Sulfoxaflor	2	2013	0.04	0.25	0.06	Reg. (EU) 2015/1295
Tebuconazole	6	2012	0.03	0.03	0.03	Dir 08/125, EFSA 08
Tebufenozide	2	2004	0.02		0.008	ESFA 10
Teflubenzuron	0.7	2017	0.01		0.016	EFSA 08
Triadimefon	0.3	2015	0.03	0.08		JMPR 2004
Triadimenol	0.3	2015	0.05	0.05	0.05	EFSA 08, Dir 08/125
Trifloxystrobin	3	2006	0.1	0.5	0.06	Reg. (EU) 2018/1060
Triflumizole	3	2014	0.05	0.1	0.05	2010/27/EU
Zoxamide	5	2008	0.5		0.3	Reg. (EU) 2018/692

**Table 2:** Pesticide identification techniques and associated advantages and drawbacks [table adapted from ref. [18,27]].

Technique	Advantages	Drawbacks
Microwave-assisted extraction (MAE)	realized easily extraction of numerous samples can occur simultaneously short extraction time required for small quantities of solvents	extraction selectivity is not sufficient need to separate extract from post-extraction residue requires clean-up step requires time for the vessels to cool down
Accelerated solvent extraction (ASE)	ability to automate extraction all process steps can be carried out identically short extraction time moderate solvent consumption preparation of sample prior to analysis is straightforward	purchasing and maintaining apparatus is costly poor extraction selectivity requires clean-up of extracts and equipment after each use which can be time-consuming
Matrix solid-phase dispersion (MSPD)	cost per analysis is relatively low straightforward equipment several analyses can be performed simultaneously can be carried out under in-situ conditions requires only small quantities of solvents	it is not possible to have a sufficiently wide analytical range in a single procedure dry samples or samples with high lipids content are not suited adsorbent consumption is relatively high requires an additional clean-up step low recoveries of analytes can occur
Solid-phase microextraction (SPME)	use of solvents can be eliminated suspended matter sensitivity is not present adsorbent capacity is limited one fibre can be utilised many times without loss of adsorbate chromatographs with ordinary injectors can be used	ensuring a sufficiently broad analytical range in a single procedure is not possible reproducibility can yield difficulties recoveries of analytes are relatively low
Supercritical fluid extraction (SFE)	substantial reduction of solvent consumption extraction of thermolabile compounds is possible analysed compounds are not degraded short extraction time relatively low labour intensity extraction permitted in semi-automatic mode with a special device	purchasing and maintaining apparatus is costly poor extraction selectivity requires clean-up of extracts and equipment after each use which can be time-consuming relatively complicated compared to other extraction techniques
Membrane extraction techniques	untreated samples introduced directly little to no use of solvent samples with very complex matrixes can be analysed high selectivity elimination of interferences analyte enrichment is high easily automated	high time consumption low efficiency membrane pores can be easily clogged by solid contaminants which leads to extended time of analysis
Liquid liquid extraction (LLE)	reliable and simple can be adapted to numerous sample types and analytes	large volume of hazardous solvent required time consuming technique
Solid phase extraction (SPE)	less time consuming than LLE purification and pre-concentration procedures are effective	requires pre-treatment requires toxic organic solvent
Traditional column-based SPE	ensures more effective sample clean-up	requires plastic cartridges containing a sorbent material and vacuum manifolds requires a larger sample requires multiple solvents, manual operation, column preconditioning, and solvent evaporation steps generates solvent waste components
Dispersive SPE (d-SPE)	recoveries of analytes with acidic or basic properties are greater and have more reproducibility uses less sorbent, sample, and equipment and thus is a cheaper and easier technique	can only be used when the SPE sorbent removes matrix components and not the analytes
Quick, Easy, Cheap, Rugged, Effective and Safe (QuEChERS)	broad scope of analytes low volume of solvents and glassware required straightforward instrumentation effective and flexible	enrichment factors are low to achieve the necessary sensitivity and thus to achieve the limits of quantification, the final extract must be concentrated to a greater extent
Dispersive liquid-liquid microextraction (DLLME)	simplicity low volume of toxic solvents rapid extraction inexpensive	low efficiency of extraction
Single Drop Microextraction (SDME)	rapid and inexpensive straightforward to operate requires little organic solvents and therefore is environmentally friendly renewability of extraction phase	less stability of the suspending drop extraction times can be lengthy
Hollow fiber-liquid phase microextraction (HF-LPME)	inexpensive volumetric ratio of the acceptor and the sample phases greatly reduced	
Continuous Flow Microextraction (CFME)	reduced solvent consumption requires inexpensive instrument rapid and straightforward efficient pesticide extraction in complex matrices	limited volume of micro-drop inserting the drop into extraction glass chamber can lead to difficulty

## Data Availability

The data presented in this study are available on request from the first author.
